# Prospective monitoring improves outcomes of primary total hip replacement: a cohort study

**DOI:** 10.1186/1754-9493-3-7

**Published:** 2009-04-14

**Authors:** Philipp N Streubel, Marcela Pachón, Carlos A Kerguelén, José Navas, Julio Portocarrero, Rodrigo F Pesantez, Gamal Zayed, Germán Carrillo, Adolfo M Llinás

**Affiliations:** 1 Departamento de Ortopedia y Traumatología, Fundación Santa Fe de Bogotá/Universidad de los Andes, Calle 119 No 9-33, Bogotá, Colombia; 2 División de Investigación, Banco de Huesos y Tejidos, Fundación Cosme y Damián, Calle 119A No 7-91, Bogotá, Colombia; 3 Centro de Gestión Hospitalaria, Carrera 11A No 94-76, Of. 201, Bogotá, Colombia

## Abstract

**Background:**

Over the past decade several studies have questioned current standards of patient safety in health care delivery. In response, our institution started a clinical pathway for total hip replacement in 1996. Prospective monitoring with regular feedback sessions to the individuals involved in patient care did however not start until 2003. The present study evaluates the effect of prospective monitoring on outcomes of total hip replacement.

**Methods:**

Clinical records of patients undergoing primary elective total hip replacement between 1997 and 2004 were reviewed. Data on adverse events as well as adherence to protocols for venous thromboembolism prophylaxis were extracted retrospectively for the period 1997 to 2001 and prospectively from 2003 to 2004. Results were compared and analyzed in order to establish possible improvement in outcomes. Data was analyzed with Chi-square or Fisher's Exact test for categorical variables and Student's t-test for continuous variables. Alpha was set as less than 5% and analysis was performed with Stata 9.0 for Macintosh.

**Results:**

Two-hundred and eighty-three patients were included from 1997 to 2001, and 62 from 2003 to 2004. Mean age, male to female ratio and initial diagnosis were similar in both groups. At least one adverse event occurred in 45% of patients in 1997–2001 and in 21% in 2003–2004 (p < 0.001). In-hospital hip dislocations occurred in 6% and 0% (p = 0.05), oliguria in 19% and 5% (p = 0.007), SSI and VTE in 3% and 0% (p = 0.37), adverse drug reactions in 11% and 13% (p = 0.66) and non-adherence to VTE prophylaxis protocols in 15% and 2% of cases respectively (p = 0.002).

**Conclusion:**

Overall rate of adverse events as well as in-hospital hip dislocations, oliguria and non-adherence to VTE prophylaxis protocols were significantly reduced during the second period. We conclude that clinical pathways alone are insufficient to improve patient safety and require prospective monitoring and continuous feedback to health care providers in order to achieve the desired effect.

## Background

The rise of malpractice claims over the last decades has lead to an increased focus on adverse events and medical error as an active field of study [1]. One large medical injury study, the Harvard Medical Practice Study, found that adverse events (AEs) occurred in 4% of hospitalizations. Furthermore, 28% of AEs were established as being secondary to negligence. Most strikingly, this study showed that 3% of AEs lead to permanent disability and 14% to a fatal outcome [2]. In a similar study focusing on surgical adverse events, Gawande et al found AEs to occur in up to 19% of surgical patients with 54% being preventable. Even though the subgroup of patients undergoing hip and knee replacement surgery had significantly fewer AEs (5%), over half of them continued to be preventable [1].

In 1999 the Institute of Medicine's (IOM) started one of the largest efforts to improve patient safety. In its report "To Err is Human", national directives for medical error prevention and detection were established [3]. Following this report, the Adult Joint Reconstruction service of our institution started a prospective monitoring program to assess adverse events and improve patient safety in the setting of primary total hip replacement (THR). As part of an earlier quality improvement project, a clinical pathway for primary THR had already been developed in 1996, prospective follow-up was nevertheless not performed from that time to 2003.

In 2003 a prospective database was therefore started on in-hospital adverse events for patients undergoing primary THR. Bi-monthly meetings were established in order to periodically present outcomes to the teams involved in delivery of care. Concurrently a retrospective review of patients undergoing primary THR from 1997 to 2001 was obtained as a control baseline.

The present manuscript compares the outcomes of the periods 1997–2001 and 2003–2004. Since no changes were introduced to the clinical pathway in the period from 1997 to 2004 we hypothesized that the active monitoring of outcomes in 2003 would lead to an initial reduction in adverse events.

## Methods

This is a prospective cohort study with a retrospective historical control group. The period of enrolment was from March 2003 to January 2004 for the prospective monitoring group and January 1997 to December 2001 for retrospective controls. Patients undergoing primary THR were included. Hip fractures and bone tumors as an indication for THR were excluded. Prospective data was obtained from patient charts, anesthesia and nursing records, and admission and follow-up notes by a physician risk manager (PNS). Bi-monthly meetings were held with individuals involved in patient care in order to discuss registered outcomes. Participants included attending physicians, residents, nurses and operation room staff. Data for the control group was retrospectively extracted by two risk management trained physicians (PNS and MP) from the same sources.

Primary outcome measures were AEs occurring during the in-hospital stay of patients undergoing primary THR. AEs were defined according to Brennan et al as injuries that occurred as a consequence to medical management and that lead to a prolonged hospital stay and/or disability at the time of discharge [2]. AEs therefore included surgical site infections (SSI), venous thromboembolic events (VTE), hip dislocations, adverse drug reactions and oliguria. In order for AEs to be registered for the study, they had to be entered by an attending or resident physician in the medical record or by a nurse in the nursing records. Oliguria was established as urinary output of ≤1 ml/kg/h according to nursing records.

No effort was done to establish the incidence of medical error for each AE. Nevertheless adherence to venous thromboembolic prophylaxis protocols was reviewed as a measure for medical error, since non-adherence represents a deviation from the accepted standard of practice dictated by the clinical pathway. For this purpose anesthesia and nursing records were reviewed and non-justified deviations from the established protocols registered.

### Surgical pathway

The procedural protocol established for THR in 1996 at our institution sets evidence based guidelines for antibiotic and VTE prophylaxis, surgical setup and rehabilitation. Antibiotic prophylaxis is started with intravenous (IV) infusion of either a first generation cephalosporin or clindamycin 20 minutes before incision and is continued QID for 24 hours or until removal of Foley catheter. VTE prophylaxis includes gradual compression stockings and low molecular weight heparin started preoperatively and continued until 35 days after surgery. Skin preparation is performed with iodophor soap and ointment and subsequent application of an iodophor impregnated adhesive. Surgery is performed under horizontal laminar flow through a posterolateral approach with capsule and external rotator reinsertion. Implants used are either total cemented or hybrid prostheses with non-cemented acetabular fixation. Femoral head sizes used during the study period were of either 22.25 or 28 mm diameter. Rehablitation following a standard protocol is routinely started on postoperative day one. All patients are discharged home with a home-based rehabilitation program.

### Statistical analysis

Chi-square and Fisher's exact test were used to compare categorical variables between groups. Student's t-test was used to compare continuous data. Alpha-level of significance was defined as less than 5% (p ≤ 0.05). Stata 9.0 for Macintosh (StataCorp LP, College Station, TX) software was used for the statistical analysis. Text for this section.

## Results

The historical control group included 283 patients with a mean age of 62 years (range 23–86). Two-hundred and six patients (73%) were female and the most common indication for surgery was osteoarthritis. Procedures were performed by a total of seven orthopedic surgeons, with an average of 10 THRs per surgeon per year. Procedure volume per surgeon ranged from 3 to 50 THRs per year. A total of 127 patients (45%) had at least one AE. The most frequent AE were oliguria in 54 patients (19%), adverse drug reactions in 31 (11%) and postoperative hip dislocations in 17 patients (6%). VTE and SSI occurred in 8 patients (3%) each. Non-adherence to VTE prophylaxis protocols was registered in 46 cases (15%).

Sixty-two patients with an average age of 68 years (range 45–90) underwent unilateral primary THRs during the active monitoring study phase. Forty-five patients (73%) were female and the most common indication for surgery was osteoarthritis. Procedures were performed by a total of five orthopedic surgeons, with an average of 12 THRs per surgeon per the evaluated 10 month period. Procedure volume during this time ranged from 2 to 52 THRs per surgeon. At least one AE occurred in 13 patients (21%). The most frequent AEs after monitoring had been started were adverse drug reactions in 8 patients (13%) and oliguria in 3 patients (5%). No hip dislocations, infections or VTEs were registered during this period. Non adherence to VTE prophylaxis protocols occurred in 1 patient (2%). Demographics and indications for surgery are summarized in table 1.

**Table 1:** Demographics, indications and outcomes

	**Monitoring (n = 62)**	**Control (n = 283)**	**p-value**
**Age **†	68 (45–90)	62 (23–86)	0.68
Gender *			
Female	45 (73)	206 (73)	0.94
Male	17 (27)	77 (27)	
Indication *			
OA	52 (84)	220 (78)	0.56
RA	4 (6)	34 (12)	
AVN	4 (6)	16 (6)	
DDH	2 (3)	13 (5)	
Adverse Event *			
Any AE	13 (21)	127 (45)	<0.001
Hip dislocation	0 (0)	17 (6)	0.05
Oliguria	3 (5)	54 (19)	0.007
SSI	0 (0)	8 (3)	0.37
VTE	0 (0)	8 (3)	0.37
ADR	8 (13)	31 (11)	0.66
VTEPP adherence *	61 (98)	241 (85)	0.002

Abbreviations: OA: osteoarthritis; RA: rheumatoid arhtritis; AVN: avascular necrosis of the femoral head; DDH: developmental displasia of the hip; AE: adverse event; SSI: surgical site infection; VTE: venous thromboembolic event; ADR: adverse drug reaction; VTEPP: venous thromboembolic event prophylaxis protocol; † values as means in years, ranges in parentheses; * values as number of patients, percentages in parentheses.

The two groups were similar with regards to age, gender and indication for surgery (p values 0.68, 0.94, and 0.56 respectively). A significant reduction in overall AE occurrence from 45% in the control group to 21% in the active monitoring group was found (p < 0.001). Incidence of hip dislocation was reduced from 6% to 0% (p = 0.05). Oliguria fell from 18.5% to 5% (p = 0.007), while SSI and VTE were reduced from 3% to 0% (p = 0.37). Non-adherence to VTE prophylaxis protocol went from 15% to 1.6% (p = 0.002). Adverse drug reaction increased from 11% to 13%, this difference was nevertheless not statistically significant (p = 0.66). Results are summarized in table 1.

## Discussion

Clinical pathways have been developed to improve effective resource use and preserve quality of care without compromising patient satisfaction or safety. Furthermore pathways have shown to reduce hospital length-of-stay [4]. Even though a clinical pathway was developed at our institution in 1996, no active monitoring had been put in place before 2003. Our retrospective review showed an unacceptably high AE incidence of 45% during the period 1997–2001. THR specific AE included hip dislocations in 6%, and VTE and SSI in 3% and 3% of patients respectively. In contrast, Philipps et al found dislocations to occur in 4%, deep SSI in 0.2% and pulmonary embolism in 0.9% of patients [5]. Their follow-up period of six months is substantially longer than the in-hospital stay reviewed for our study. In a nationwide study Zhan et al reviewed short term outcomes of patients undergoing THR in 2003. In-hospital mortality was found to be 0.33%, while VTE occurred in 0.68%, decubitus ulcers in 0.28%, postoperative hemorrhage or hematoma in 0.05% and infection in 0.05% [6]. Lassen et al reviewed the occurrence of symptomatic venous thromboembolism at 11 days after elective THR. They found a 0.3% incidence using a VTE prophylaxis regimen of enoxaparin 40 mg subcutaneously started preoperatively [7]. This regimen is similar to the one used at our institution and is therefore a useful reference point. By these means, before starting prospective monitoring of our pathway, frequencies were unacceptably high for each of the listed AEs.

After starting active monitoring, overall rate of adverse events as well as in-hospital hip dislocations, oliguria and non-adherence to VTE prophylaxis protocols significantly decreased. THR specific AEs even reached values similar to those reported in the literature. Since no changes were introduced to the clinical pathway during the periods included in this study, the most likely explanations for outcome improvement is the introduction of active monitoring and feedback to the actors of delivery of care. The phenomenon of increased performance derived from the perception of being observed has been an extensive field in industrial psychology. The landmark studies on productivity at the Western Electrical Company's Hawthorne Works in Chicago during the 1920s and 30s lead to the finding that worker's performance increased as a consequence to being singled out or to be made to feel important [8]. As a consequence the term "Hawthorne Effect" (HE) has been coined as an increase of an individual's productivity secondary to an expression of interest in that individual's performance [9].

In our study, the presence of an individual, in our case a risk manager, prospectively recording patient AEs and outcomes may have increased the alertness towards unsafe practices. The feedback offered through bi-monthly meetings may have further strengthened this effect. In this sense a surgeon who is aware of hip dislocation rates being measured and communicated to all team members will be prone to make an additional effort in obtaining the best operative results. Even though several factors not controllable by the surgeon certainly influence the risk of postoperative hip dislocation, several others, including soft tissue tension and prosthetic alignment clearly depend on the surgeon's task. Furthermore, nurses and therapists will put an additional effort in avoiding maneuvers that may put the patient's hip stability at risk. This same reasoning may hold true for the reduction of oliguria, VTE and SSI as well as improvement of VTE prophylaxis protocol adherence.

One further remarkable finding of our study was the 15% prevalence of non-adherence to VTE prophylaxis protocols. This is of special importance, since protocols follow evidence based management guidelines aimed at adverse event reduction. Unjustified non-adherence to such a protocol would parallel not using a seat belt while driving an automobile. While it does not always result in an unfavorable outcome, it puts the patient at an unnecessary risk[10]. Even though our study did not show improved adherence to lead to a significant reduction of VTE, we believe that a larger sample size would have shown a significant effect. In this sense prospective monitoring should have a strong impact on patient safety as it increases protocol adherence as shown by the almost 10-fold increase in VTE prophylaxis protocol adherence in our study.

One limitation of our study is the relatively small sample size of the prospective monitoring group. In January 2004 multiple modifications, like intraoperative intermittent neumatic compression for VTE prophylaxis and use of larger femoral heads, were introduced in the clinical pathway. Since these changes would have altered the prospective group's homogeneity and hence its comparability with the retrospective control group, we included only patients operated before their introduction.

## Conclusion

Even though the purpose of clinical pathways is to improve effective resource use and increase patient satisfaction, our study suggests that without active monitoring patient safety can not be warranted. Prospective follow-up of outcomes and regular follow-up to health care provider is essential to reduce adverse events and improve patient safety.

## Competing interests

The authors declare that they have no competing interests.

## Authors' contributions

PNS and MP participated in study conception and design, performed data extraction and monitoring and performed statistical analysis. AML, CAK and JN participated in study design and helped to draft the manuscript. JP, RFP, GZ and GC conceived and coordinated the study and did final manuscript revision. All authors read and approved the final manuscript.

## Authors' information

PNS: Six Sigma Black Belt, Orthopaedic Surgeon. JN: Orthopaedic Surgeon, Chief of Reconstructive Hip Surgery, Department of Orthopaedic Surgery, Fundacion Santa Fe de Bogotá, and Director Fundación Cosme y Damián. CAK: Consultant for Risk Management, Centro de Gestión Hospitalaria. JP: Director, Centro de Gestión Hospitalaria. RFP, MP, GZ, GC: Orthopaedic Surgeon. AML: Orthopaedic Surgeon, Chairman Department of Orthopaedic Surgery, Fundación Santa Fe de Bogotá, and Research Consultant Fundación Cosme y Damián.
